# A Mason-Pfizer Monkey Virus Gag-GFP Fusion Vector Allows Visualization of Capsid Transport in Live Cells and Demonstrates a Role for Microtubules

**DOI:** 10.1371/journal.pone.0083863

**Published:** 2013-12-26

**Authors:** Jasmine Clark, Petra Grznarova, Elizabeth Stansell, William Diehl, Jan Lipov, Paul Spearman, Tomas Ruml, Eric Hunter

**Affiliations:** 1 Emory Vaccine Center at the Yerkes National Primate Research Center, Emory University, Atlanta, Georgia, United States of America; 2 Department of Biochemistry and Microbiology, Institute of Chemical Technology, Prague, Czech Republic; 3 Department of Pediatrics, Emory University School of Medicine, Atlanta, Georgia, United States of America; 4 Department of Pathology, Emory University, Atlanta, Georgia, United States of America; NCI-Frederick, United States of America

## Abstract

Immature capsids of the Betaretrovirus, Mason-Pfizer Monkey virus (M-PMV), are assembled in the pericentriolar region of the cell, and are then transported to the plasma membrane for budding. Although several studies, utilizing mutagenesis, biochemistry, and immunofluorescence, have defined the role of some viral and host cells factors involved in these processes, they have the disadvantage of population analysis, rather than analyzing individual capsid movement in real time. In this study, we created an M-PMV vector in which the enhanced green fluorescent protein, eGFP, was fused to the carboxyl-terminus of the M-PMV Gag polyprotein, to create a Gag-GFP fusion that could be visualized in live cells. In order to express this fusion protein in the context of an M-PMV proviral backbone, it was necessary to codon-optimize *gag*, optimize the Kozak sequence preceding the initiating methionine, and mutate an internal methionine codon to one for alanine (M100A) to prevent internal initiation of translation. Co-expression of this pSARM-Gag-GFP-M100A vector with a WT M-PMV provirus resulted in efficient assembly and release of capsids. Results from fixed-cell immunofluorescence and pulse-chase analyses of wild type and mutant Gag-GFP constructs demonstrated comparable intracellular localization and release of capsids to untagged counterparts. Real-time, live-cell visualization and analysis of the GFP-tagged capsids provided strong evidence for a role for microtubules in the intracellular transport of M-PMV capsids. Thus, this M-PMV Gag-GFP vector is a useful tool for identifying novel virus-cell interactions involved in intracellular M-PMV capsid transport in a dynamic, real-time system.

## Introduction

In studying the role of viral and host cell factors involved in retroviral assembly, trafficking, and budding, the mechanism by which capsids are transported through the cytoplasm is still an poorly understood process. It has been shown previously that for Mason-Pfizer Monkey Virus (M-PMV), capsids are assembled intracellularly at the pericentriolar region by way of a cytoplasmic targeting-retention signal (CTRS), and are subsequently trafficked to the plasma membrane for budding [1,2]. This process is unique when compared to a virus such as HIV, which exhibits C-type retrovirus morphogenesis, in that assembly and budding are spatially and temporally separated. This separation thus allows for studying these processes individually in order to elucidate the role of viral and cellular factors necessary for assembly and intracellular transport. 

Like most simple retroviruses, M-PMV encodes a Gag polyprotein, Pr78, that assembles to form the immature capsid and expression of this gene alone results in the assembly and release of immature virus particles [3]. Pr78 is modified by an N-terminal myristic acid residue that is mostly buried within the folded protein [4]. Following interaction of Gag with the plasma membrane the exposure of this myristic acid moiety appears to be critical for envelopment and budding of the virus [5]. These previous studies with M-PMV have employed the use of genetic, biochemical and fixed-cell immunofluorescent methodologies for elucidating the role of viral and cellular components in viral assembly, transport, and budding [2,6-9]. Prior data has shown that blocking vesicular trafficking by shifting the temperature of the cell to 20°C causes a significant delay in M-PMV Gag release kinetics and maturation, indicating a role for the cellular vesicular transport system in capsid transport [10,11]. It has also been shown that Env glycoprotein expression is necessary for efficient capsid release and maturation [7,10,12]. Several capsid mutations have provided key insights into the processes involved in M-PMV assembly and transport. Specifically, a single amino acid change from arginine to tryptophan (R55W) in the CTRS causes a switch from B/D type capsid assembly to C-type assembly [3] by preventing nascent Gag molecules from interacting with the dynein light chain Tc-tex and their subsequent transport on microtubules to the pericentriolar region of the cell. Another mutation in the matrix (MA) protein of Gag, R22A, displays a transport defect in which assembled immature capsids are unable to reach the plasma membrane resulting in their aggregation under the cortical actin layer. This dominant negative mutation, which appears to block transit through the actin layer, causes a complete block in viral release from the cell [5]. Further, a double mutation, K16A/K20A, in a basic region of MA, results in the budding of immature capsids into intracellular vesicles, suggesting that these residues play an important role in regulating myristic acid exposure . These data and mutants, while providing insights into the dynamics of Gag assembly, intracellular trafficking, and interactions with cellular membranes, have not allowed studies of individual capsid movement within the cell. 

 Recent advances in molecular virology have allowed for the use of fluorescently tagged proteins for studying intracellular viral processes. In these studies, a fluorescent protein, usually enhanced green fluorescent protein (eGFP), is fused to the viral protein of interest followed by utilization of real-time live cell imaging techniques for visualizing its behavior. In fusing GFP to the protein of interest, however, several controls are required in order to make sure that the fusion does not disrupt the normal biological processes of the protein. For HIV, Muller et al., were able to successfully tag HIV Gag by inserting GFP near the C-terminus of the matrix (MA) domain of Gag, which had previously been shown to accept short epitope tags without disrupting replication [13]. Successful fusion of GFP to viral capsid proteins, has been used for the study of virus intracellular localization, assembly, and trafficking for other viruses such as herpes simplex virus (HSV) and adeno-associated virus (AAV) [14,15]. Therefore, construction of a GFP-tagged protein that functions similarly to the wild-type counterpart, can be an integral tool in studying viral-host cell interactions in a dynamic system. 

In this study, we set out to create an M-PMV Gag-GFP fusion construct that could be used to investigate capsid assembly and the role of the cytoskeleton in intracellular trafficking in a dynamic system. In order to achieve this, it was necessary to codon-optimize the M-PMV *gag*, strengthen the Kozak consensus sequence for the initiating methionine of Gag, mutate an internal initiating methionine codon of *gag* and replace the M-PMV *pro* and *pol* genes with the gene for eGFP. Cotransfection of this Gag-GFP vector with a helper provirus resulted in capsid assembly and release with an efficiency similar to WT M-PMV. The intracellular localization of eGFP-tagged WT and mutant constructs was compared to untagged proteins labeled by immunofluorescent staining in fixed cells and shown to be equivalent. Fluorescently-labeled, mobile capsids were visualized in live cells, and both the kinetics and co-localization of capsids show a key role for microtubules in their intracellular transport from the pericentriolar region to the plasma membrane.

## Materials and Methods

### Cell Lines

Infectious M-PMV-producing CMMT cells, were originally derived by co-culturing rhesus mammary tumor cells with rhesus monkey embryo cells [16-18]. COS-1 cells, derived from the African green monkey kidney cell line, CV-1, by transformation with an origin-defective mutant of SV40 [19] and 293T cells, derivatives of the 293 cell line containing an insertion of the temperature sensitive gene for the SV40 T-antigen [20], were obtained from the American Type Culture Collection. All cells were cultured in Dulbecco’s modified Eagle’s medium (DMEM) supplemented with 10% fetal bovine serum (Gibco). Cell lines were maintained at 37°C with 5% CO_2_.

### Plasmids

**Figure 1:**
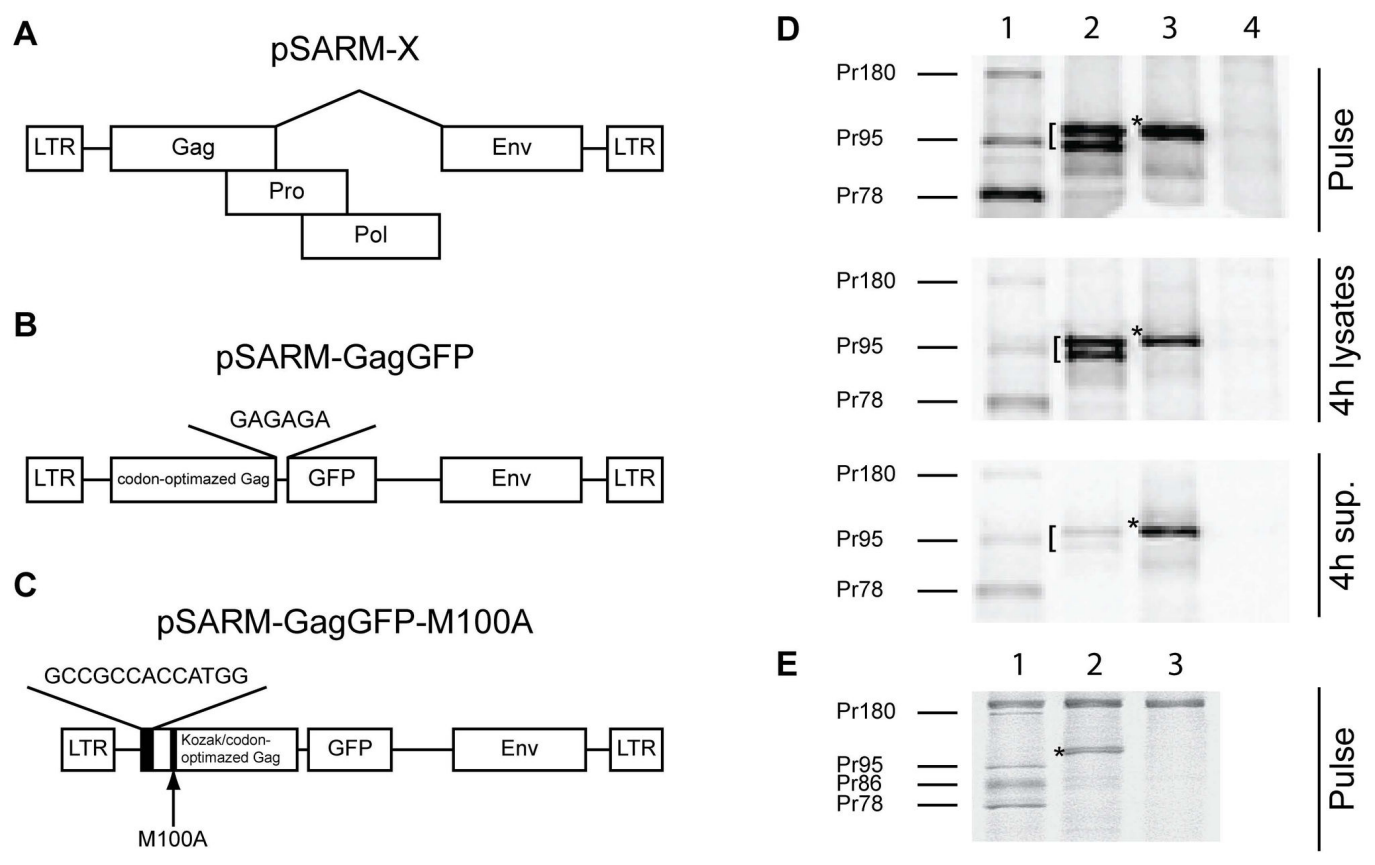
Construction of GagGFP pSARM-X. Genetic organization of construct pSARM-X (A), pSARM-GagGFP (B), pSARM-GagGFP-M100A (C). (D) 293T cells (untransfected, Lane 4) were transfected with pSARM-D26N (Lane 1), pSARM-GagGFP (lane 2) and pSARM-GagGFP-M100A (Lane 3). Viral proteins were metabolically labeled with [^35^S]-methionine followed by a 4-hour chase in unlabeled media. Lysates from the pulse and chase (4h lysates) as well as culture medium from the chase (4h sup.) were then immunoprecipitated with rabbit anti-p27^CA^ antibody and resolved on SDS-PAGE. (E) 293T cells were transfected with pSARM-D26N (Lane 1) and pSARM-GagGFP-M100A (Lane 2). Viral proteins were metabolically labeled with [^35^S]-methionine and lysates were then immunoprecipitated with goat anti-MPMV antibody and resolved on SDS-PAGE. Positions of the viral precursor proteins Pr180 (Gag-Pro-Pol), Pr95 (Gag-Pro), Pr86 (Envelope) and Pr78 (Gag) are shown. Products of pSARM-GagGFP ([) and pSARM-GagGFP-M100A (*) are shown.

The plasmids used in this study are depicted in Figure 1. The plasmid pSARM-X is an M-PMV proviral vector that expresses the M-PMV genome under the control of the viral LTRs (Figure 1A). The plasmid pSARM-GagGFP was constructed by inserting a codon-optimized *gag* gene and linked e*gfp* gene between the EagI and XhoI sites of pSARM-X, replacing the *gag, pro*, and *pol* genes. Briefly, the codon-optimized *gag* gene was amplified with primers containing an EagI site in the forward primer and an AgeI site in the reverse primer. The eGFP was amplified from a pEGFP-N1 vector using primers with an AgeI site in the forward primer and a PspXI site and putative splice acceptor site in the reverse primer. The amplified *gag* was digested with EagI and AgeI; the amplified *eGFP* was digested with AgeI and XhoI (and isoschizomer of PspXI); and the pSARM-X provirus was digested with EagI and XhoI. Fragments were ligated by three-way ligation to create pSARM-GagGFP (Figure 1B). The vector was confirmed using both diagnostic digestion with BlpI and sequencing of the complete insert. 

To enhance the Kozak consensus sequence of the Gag-GFP construct and to prevent internal ribosomal initiation, the plasmid was mutated using four overlapping ultramers spanning from the EagI site to the SbfI site of codon-optimized *gag*. The ultramers contained a strengthened Kozak consensus sequence at the initiating methionine as well as a methionine to alanine substitution at the second methionine (M100A) (Figure 1C). All four ultramers were annealed using temperature-gradient PCR (50-65 °C) with Taq polymerase. The subsequent 500 bp fragment was digested with EagI and SbfI and inserted back into pSARM-GagGFP to create pSARM-GagGFP-M100A.

The M-PMV proviruses encoding the R55W, -R22A, -K16A/K20A mutations have been described previously [3,5]. The GagGFP-M100A-R55W, -R22A, -K16A/K20A mutants were derived using the QuikChange Lightning Site-directed Mutagenesis Kit (Agilent Technologies, La Jolla, CA), using pSARM-GagGFP-M100A as the template and respective mutagenic primers (Table 1). Mutants were confirmed by sequencing.

**Table 1:** List of primers.

**Plasmid**	**Primer Name**	**Primer Sequence**
pSARM-GagGFP	GagGFP F-EagI	5'-TGCGTCTCAACCTCGGCCG
	GagGFP R-AgeI	5'-CGACCGGTCCCGCACCTTCATACTGTGTTGGAGG
	GFP F-AgeI	5'-TGCCTCCTCCAACACAGTATTGAGTGCGGGACCGGT
	GFP R-PspXI + SA	5'-ACTCTCCTCGAGGAGAGAATCACTTGTACAGCTCGTC CATGCCGA
PSARM-GagGFP-M100A	Kozak F-EagI ultramer	5'-CAACCTCGGCCGGCGATTAAAAGTGAAAGTAAACTCTCTTGGCCGCCGCGGGAACCTGCCGCGTTGGACCTGAAAGT AAGTGTTGGCCGCCACCATGGGACAGGAGCTGTCACAGCATGAGAGGTATGTGGAGCAGCTGAAGCAG
	Kozak R ultramer	5'-GAAAGCAATCTCCCACTCTTCTCCAGCGCTTAATGTCGATGGTTCCCTCTTGGGGAAACCAAGGACAGGTGTCTTTT ACGAAATCAAAAAATTTCAGAAGGTCGGCGTATTTGACCTTCACTCCCCTGGTTTTAAGGGCCTGCTTCAGCTGCTCCACATA
	M100A F ultramer	5'-GCTGGAGAAGAGTGGGAGATTGCTTTCAGGACTACTATAACACATTTGGCCCCGAAAAAGTTCCCGTTACTGCATTT AGCTACTGGAATCTCATTAAAGAGCTGATTGATAAAAAGGAAGTGAACCCACAGGTCGCTGCCGCAGTCGCGCAGACTGAG
	M100A R-SbfI ultramer	5'-CTTTGTCCTGCAGGGAAGAAGACTTGGCTCCTTCGTCGTCACTATCCAGAGAAATAAGGTCCAAGTCGGGATTCTGT GATGTCTTCGTCAGGTCCGTCTGACTATTGCTTTTAAGAATTTCCTCAGTCTGCGCGACTGCGG
pSARM-GagGFP-R55W	R55W F	5'-GGAACCATCGACATTAAGTGGTGGAGAAGAGTGGGAGA
	R55W R	5'-TCTCCCACTCTTCTCCACCACTTAATGTCGATGGTTCC
pSARM-GagGFP-K16A/K20A	K16A/K20A F	5'-CATGAGAGGTATGTGGAGCAGCTGGCGCAGGCCCTTGCGACCAGGGGAGTGAAGGTCAAATAC
	K16A/K20A R	5'-GTATTTGACCTTCACTCCCCTGGTCGCAAGGGCCTGCGCCAGCTGCTCCACATACCTCTCATG
pSARM-GagGFP-R22A	R22A F	5'-GCCCTTAAAACCGCGGGAGTGAAGGTC
	R22A R	5'-GACCTTCACTCCCGCGGTTTTAAGGGC

### Metabolic Labeling and Immunoprecipitation

A total of 4 μg of the various pSARM-derived constructs were transfected into COS-1 or 293T cells using Lipofectamine 2000 (Invitrogen, Carlsbad, CA). At 18-24 hours after transfection, cells were starved in DMEM lacking methionine and cysteine (Gibco) for 15 minutes. Cells were subsequently labeled with 100 µCi ^35^S-methionine (Perkin-Elmer NEN, Boston, MA) per well for 30 minutes. Following labeling (Pulse), the labeling media was replaced with complete DMEM (Gibco) and cultured for the indicated times (Chase). Cell-associated and cell-free viral proteins were then analyzed. Cell supernatants were collected from each well and cellular debris was removed by low-speed centrifugation and virus particles lysed by the addition of Triton X-100 to 1%. Cells in each well were subsequently gently lysed with 1% Triton X-100 lysis buffer [1% Triton X-100, 50mM NaCl, 25mM Tris, pH 8.0]. Cell lysates were pre-cleared with fixed *Staphylococcus aureus* (*Staph. A.*) overnight at 4°C, then M-PMV proteins were immunoprecipitated using either polyclonal goat-anti-M-PMV or rabbit-anti-Pr78 anti-sera. Immune complexes were isolated by addition of fixed *Staph. A*. and washed with lysis buffer containing 0.1% SDS (0.1% SDS, 1% Triton X-100, 50mM NaCl, 25mM Tris, pH 8.0; lysis buffer B) [21]. The immunoprecipitated proteins were resolved by electrophoresis on 12% SDS-polyacrylamide gels (SDS-PAGE) (BioRad).

### Quantifying Gag Release

SDS-PAGE gels were fixed in Coomassie Blue solution [0.05 % Coomassie Brilliant Blue, 10% glacial acetic acid, 50% methanol, 40% H_2_O], dried and then exposed to phosphor screens and the radiolabeled protein bands were digitally acquired using the Packard Cyclone**™** system. Optiquant imaging analysis software (Perkin Elmer, Shelton, CT) was used to quantify the amount of viral proteins based on the number of digitized light units (DLU) for each band. To measure the percentage of M-PMV GagGFP released, the ratio of GagGFP in the supernatant versus total cell-associated and released GagGFP was determined. Conversely, to measure the percentage of cell-associated M-PMV Gag, the ratio of total cell-associated Gag versus total cell-associated and released Gag was determined. For the positive control, the total cell-associated and released Gag (Pr78, Pr95, and Pr180) was measured. 

### Electron Microscopy

COS-1 cells were transfected with the indicated plasmids using Fugene 6 (Promega) transfection reagent. At 24 hours post-transfection, cells were fixed in a 2.5% glutaraldehyde fixative (10%- 25% EM grade glutaraldehyde, 50% 0.2M cacodylate buffer, 40% distilled H_2_0) for at least two hours at 4°C. The cells were washed and subsequently fixed in 1% osmium fixative (2 parts 0.2M cacodylate buffer, 1 part 6% potassium ferrocyanide, 1 part 4% osmium tetroxide). The cells were then dehydrated with ethanol, followed by infiltration with a 1:1 ratio of 100% ethanol and Epon resin for at least one hour. Cells were embedded in fresh Epon resin at 60°C for 48 hours, and then cut into ultrathin sections and picked up on a copper grid. Virus particles were viewed using a JEOL-JEM 1210 transmission electron microscope.

### Virus Purification

293T cells seeded onto a 10 cm culture plate were transfected with 15 μg of total DNA with Lipofectamine 2000. For co-transfected cells, a ratio of approximately 4:1 (11.5 μg and 3.5 μg of pSARM-GagGFP-M100A and pSARM-D26N, respectively) was used. Proteins in transfected cells were radiolabeled with ^35^S for 48 hours by incubating cells in 90% Met-/Cys- DMEM (Gibco) in complete DMEM complemented with 100 μCi/mL ^35^S. Cellular supernatants were filtered through a 0.45 um filter (Thermo-Scientific, Waltman, MA) then overlaid on a 20%-50% sucrose (w/w), with 10% steps, discontinuous gradient. Samples were spun at 35,000 rpm at 4°C for 3 hours in the Beckman-Coulter Optima L-80 XP Ultracentrifuge using the SW41-Ti swinging bucket rotor. Using upward displacement, 1 mL fractions were isolated. 200 μl of Lysis Buffer A and 6 μl of 20% SDS were added to each fraction, and virions were immunoprecipated with a polyclonal goat-anti-M-PMV antisera. Immune complexes were isolated by addition of fixed *Staph. A*. and washed with lysis buffer B. Immunoprecipitated proteins were resolved on 12 % SDS-PAGE gels. The density of each fraction was calculated using refractive indices.

### Western Blot

Supernatants from 10cm culture dishes of 293T cells transfected with 4μg of pSARM-GagGFP-M100A or 1.6 μg and 3.2 μg pSARM-GagGFP-M100A and pSARM-X, respectively, were collected 48h post transfection. Virions were isolated by ultracentrifugation through a 25% sucrose cushion and the pellet was resuspended in 100 μl protein loading buffer. 25 μl of each sample was resolved in a 12% SDS-PAGE gel. Samples were transferred to a nitrocellulose membrane, blocked with 5% non-fat dry milk, and blotted with a mixture of two monoclonal mouse-anti-GFP antibodies (Roche, Germany). Immune complexes were subsequently bound by a horseradish peroxidase (HRP)-conjugated donkey-anti-mouse IgG antibody (Santa Cruz Biotechnology, Dallas, Texas). Pierce SuperSignal West Femto Chemiluminescent HRP substrate (ThermoScientific, Rockford, IL) was applied to blot followed by exposure to film.

### Fixed Cell Microscopy

For imaging GFP-tagged as well as untagged Gag, COS-1 cells were plated at 80-90% confluency on coverslips (Fisher Scientific). Cells were co-transfected at a ratio of 4:1 with 3.2 μg WT or mutant M-PMV provirus and 0.8 μg WT or mutant pSARM-GagGFP-M100A, or were transfected with the untagged WT or mutant provirus only. 24 hours post transfection, cells were fixed with cold methanol:acetone (1:1). Coverslips containing cells transfected with GFP-tagged virus were then placed on glass slides with Prolong Antifade Gold (Invitrogen). Coverslips containing the fixed cells transfected with untagged provirus were reacted with polyclonal rabbit-anti-p27 antisera (1:500 dilution), and after washing 3X in PBS, bound antibodies were tagged with 1:10,000 diluted Alexa-488-conjugated Goat-anti-Rabbit IgG (Invitrogen) and subsequently placed on glass slides with Prolong Antifade Gold. Cells were then imaged on the Deltavision Core Imaging System (Applied Precision, Issaquah, Washington) with a CoolSnap camera at 60X magnification. 

### Live Cell Microscopy

CMMT cells, which constitutively produce WT M-PMV proteins, were plated to 70-80% confluency on 35-mm coverslip-bottom dishes (Matek, Ashland, MA), then transfected with 4 μg pSARM-GagGFP-M100A construct using the Lipofectamine 2000 transfection reagent. For co-localization studies with microtubules, cells were co-transfected with pSARM-GagGFP-M100A and p-mCherry-tubulin (A gift from Roger Tsien to Paul Spearman). Images were acquired using the Deltavision Core Imaging System with a CoolSnap camera. All experiments were done in a 37°C chamber with CO_2_ infusion at 60X magnification. 

### Time-lapse Image Analysis

Deltavison (.dv) files were imported into FIJI, an NIH open-source image analysis software distribution, using the Bio-Formats Importer (LOCI) plugin [22,23]. Particles were detected and tracked in two- and three dimensions using the Particle Tracker (MOSAIC) plugin. This plugin allowed for automated tracking of single particles based on an algorithm described by Sbalzarini et al. [24]. To calculate maximum instantaneous velocity, the instantaneous velocity at each time point was calculated using the x- and y- position of a 2D image and the following formula:

$vn=\frac{\sqrt{{\left(x_{n+1}-x_{n}\right)}^{2}+{\left(y_{n+1}-y_{n}\right)}^{2}}}{t}\times pixel\_size$

v_n_ - instaneous velocity at time *n*
x_n_ - x-position at time *n*
y_n_ - y position at time *n*
t - time lapse between successive frames

pixel size - representative size/pixel

To calculate the total displacement of a trajectory, the distance between the beginning and end of a trajectory was calculated using the x- and y- position of the 2D image and the following formula: 

$d_{total}=\sqrt{{\left(x_{last}-x_{first}\right)}^{2}+{\left(y_{last}-y_{first}\right)}^{2}}$

d_total_- total displacement
x_first_- x-position at beginning of trajectory
x_last_- x-position at end of trajectory
y_first_- y-position at beginning of trajectory
y_last_- y-position at end of trajectory

For tracking along microtubules, single particles were manually tracked by following the particle in successive frames until the particle was no longer visible or no longer able to be discriminated by eye. To define trajectory types, trajectories were categorized by visual examination of the trajectories made using the ImageJ Particle Tracker (MOSAIC) plugin. Trajectories with less than (<) 10 frames were filtered out. For quality control, the total displacement of the trajectories was calculated.

## Results

### Construction of an M-PMV provirus expressing a Gag-eGFP fusion protein

Initial experiments to construct a GFP-tagged M-PMV Gag protein, in which the *pro-pol* region of the genome was replaced with the gene for eGFP, resulted in the synthesis of a ~70kD protein consistent with the product of aberrant splicing (Data not shown). We therefore codon-optimized the *gag* gene and created a *gag*-*egfp* gene fusion as described in Methods. In order to express the codon-optimized Gag-GFP fusion protein from an M-PMV provirus, the chimeric gene was inserted into pSARM-X to replace the *gag, pro* and *pol* genes as described in Methods (Figure 1A, B). Since the *pro* and *pol* genes were removed from this construct and cleavage could not occur upon release, a pSARM-X construct containing a mutation in the active site of *pro*, D26N, was used as the positive control in the experiments described below. 

In order to investigate whether the Gag-GFP construct was synthesized, assembled, and transported with similar kinetics to WT Gag, pulse-chase experiments were carried out. Immunoprecipitation of pulse-labeled cells transfected 24 hours previously with the pSARM-D26N plasmid showed major bands at 78, 95, and 180 kD (Figure 1D, Lane 1 Pulse), consistent with the Gag, Gag-Pro, and Gag-Pro-Pol precursors. Pulse-chase analysis of the new construct, pSARM-GagGFP showed two major bands at approximately 95 and 105 kD (Figure 1D, Lane 2 Pulse). Both of the bands were inefficiently released into the supernatant after a 4h chase in complete medium (Figure 1D, Lane 2 4h Sup). 

An analysis of the sequence of the codon-optimized *gag* gene showed a weak Kozak-consensus sequence preceding the initiating methionine, as well as a second in-frame methionine at amino acid 100 (M100), raising the possibility that ribosomes, were traversing the primary initiating methionine of *gag* and initiating translation at M100. We therefore optimized the Kozak consensus sequence at the initiating methionine, and substituted an alanine codon for that of M100 (M100A) (Figure 1C and Figure S1). M-PMV M100A had previously been shown to have no effect on Gag processing or release kinetics [9]. The Kozak-optimization and the M100A mutation in this pSARM-GagGFP-M100A construct resulted in efficient initiation from the first methionine, eliminated expression of the truncated protein, and showed Gag-GFP expression at comparable levels to D26N (Figure 1D, Lane 3 Pulse). Moreover, these modifications resulted in more efficient transport and release of the Gag-GFP fusion protein (Figure 1D, Lane 3 4h sup.).

A pulse-chase analysis also revealed that the Env glycoprotein was inefficiently expressed in the Gag-GFP fusion construct even though the *env* gene was present. In wild-type M-PMV Env is expressed from a spliced mRNA, and all putative *env* splice acceptor sites (Figure S1) are present in the region flanking the 3’ end of GFP in the pSARM-GagGFP-M100A fusion, but this construct still yielded background levels of Env expression (Figure 1E). Analysis of the nucleotide sequence of non-codon-optimized versus codon-optimized M-PMV Gag showed that a majority of the potential splice branch points were removed during codon-optimization (data not shown). The possibility that the branch point for Env splicing was in the *pro* or *pol* genes, which have been completely removed from this construct, also cannot be ruled out. 

### Expression of eGFP-fused Gag alone causes aberrant capsid formation

**Figure 2:**
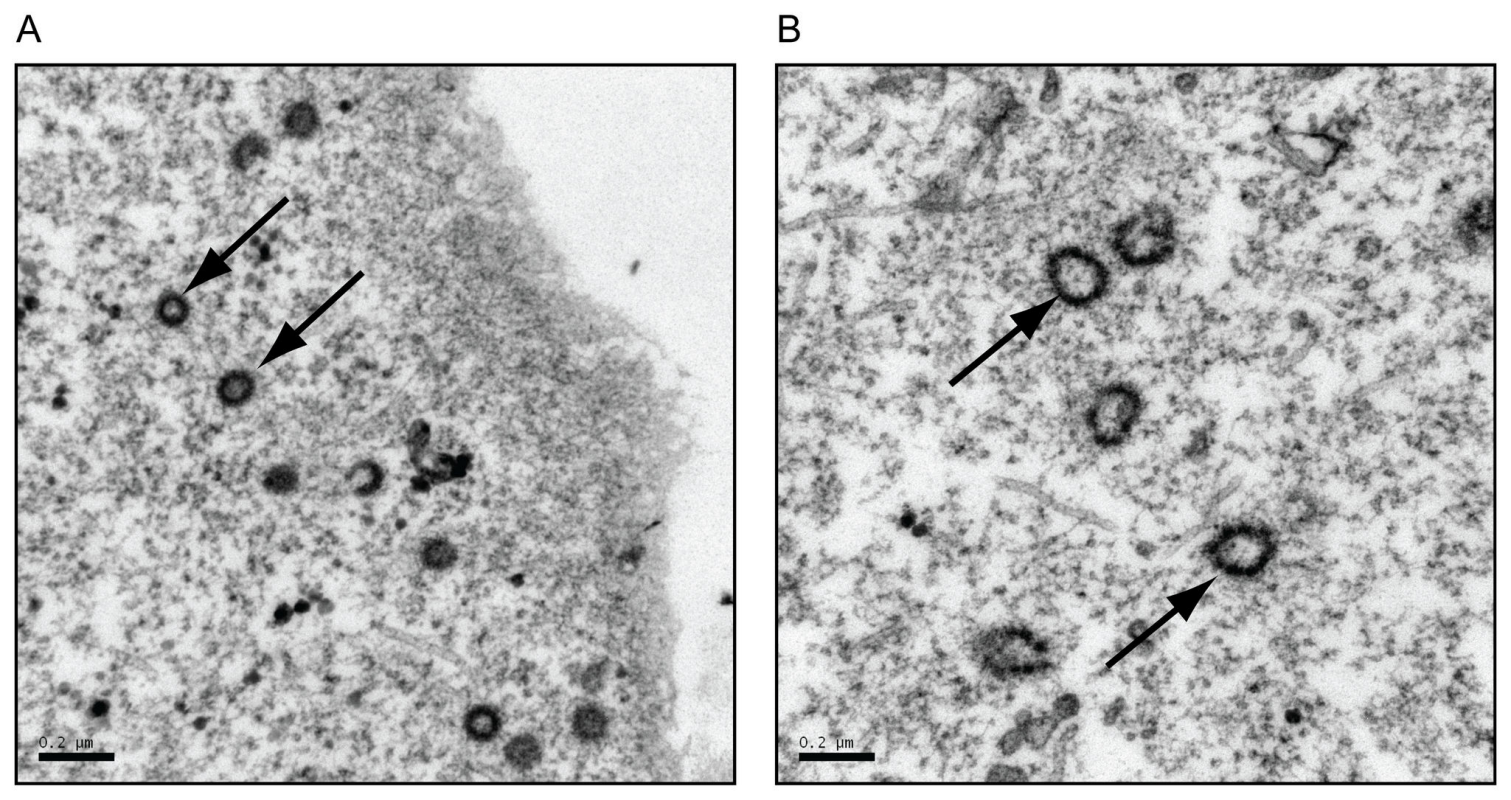
Verification of viral particles shapes by transmission electron microscopy. COS-1 cells were transfected with pSARM-X wild type provirus (A) or pSARM-GagGFP-M100A (B). 24 hours after transfection were cells fixed in a 2.5% glutaraldehyde fixative for 2 hours at 4 °C. The cells were washed and subsequently fixed in 1% osmium fixative. The cells were then dehydrated with ethanol, followed by infiltration with 100% ethanol and Epon resin for at least one hour. Cells were then embedded in fresh Epon resin at 60°C for 48 hours then cut into ultrathin sections and picked up on a copper grid. The arrows in (A) point to WT intracellular capsids of homogenous shape, with a diameter of approximately 100 nm. The arrows in (B) point to GagGFP-containing intracellular capsids having a beads-on-a-string shape and varying diameters greater than 100 nm. Bar = 200 nm.

To determine if the fusion of a 27 kD eGFP protein to the carboxyl terminus of Gag impacted the shape and size of the resulting immature capsids, capsid structure was visualized using transmission electron microscopy (TEM). 293T cells were transfected with pSARM-GagGFP-M100A or pSARM-X, and prepared for TEM. The results from analysis of thin section electron-micrographs showed that capsids from cells transfected with pSARM-GagGFP-M100A were aberrantly shaped in comparison to capsids from cells transfected with the WT provirus (Figure 2B), although they were distributed similarly throughout the cytoplasm (Figure S2A and B). The immature capsids in cells transfected with pSARM-GagGFP-M100A were larger in diameter, were not uniform in shape, and had a “beads-on-a-string” morphology compared to the more compact, homogenously-shaped immature capsids of the WT (Figure 2B versus 2A). 

### Release kinetics of capsid is delayed in the absence of WT virus and rescued during cotransfection with WT

**Figure 3:**
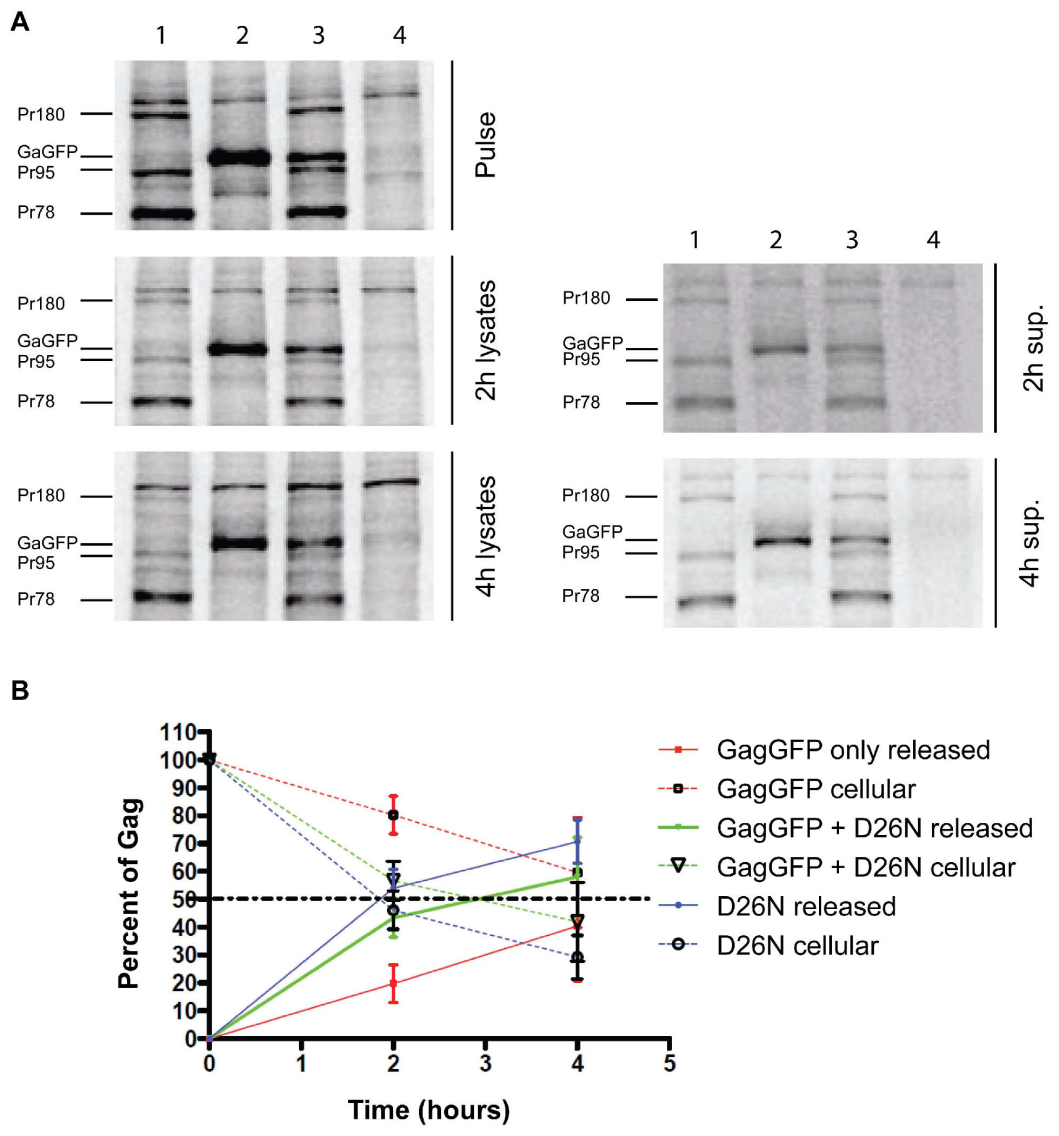
Analysis of release kinetics of the GagGFP fusion protein. (A) 293T cells (untransfected, Lane 4) were transfected with pSARM-D26N (Lane 1), pSARM-GagGFP-M100A (Lane 2) and co-transfected (Lane 3) with pSARM-D26N and pSARM-GagGFP-M100A at a 4:1 ratio. Viral proteins were metabolically labeled with ^35^S methionine and then immunoprecipitated with rabbit anti-p27^CA^ antibody from lysates of pulse-labeled cells (pulse), pulse-labeled and 2h and 4h chased cells (2h lysates and 4h lysates) and from the culture media collected after 2h or 4h chase respectively (2h and 4h sup.). Positions of the viral precursor proteins Pr180 (Gag-Pro-Pol), Pr95 (Gag-Pro) and Pr78 (Gag) and product of pSARM-GagGFP-M100A are shown. (B) The percentage of cell associated viral protein was measured by calculating the ratio of protein from the lysates versus the total protein (lysates and sup.). Conversely, the percentage of released viral protein was measured by calculating the ratio of protein from the supernatants versus the total protein. For D26N, the total amount of cell-associated and released Gag is calculated by adding the amounts of each of the precursors (Pr180, Pr95, and Pr78). For GG and GG co-transfections, the amount of Gag cellular and released Gag corresponds to the amount of Gag-GFP.

Since previous reports have shown that Env expression is necessary for efficient virion release and capsid maturation [2,7,10,12], and the TEM data showed improper capsid morphology when cells were transfected with pSARM-GagGFP-M100A alone, a pulse-chase experiment was carried out to determine whether cotransfection with WT pSARM-X changed the kinetics and efficiency of Gag-GFP release from cells. Proteins were pulse-labeled with ^35^S methionine for 30min and chased with unlabeled media for 2 or 4 hours. Cell lysates from the pulse and each chase time-point, as well as cell culture medium (supernatants) from the chase plates were immunoprecipitated with rabbit anti-p27^CA^ antibody (Figure 3A). The mutant pSARM-D26N was used as the positive control (Lane 1). Radioactivity corresponding to the immunoprecipitated proteins were quantitated as described in Methods. Consistent with previous reports, approximately 2 hours after the pulse, 50% of the cell-associated precursors (Pr78, Pr95, and Pr180) had been released into the culture medium (Figure 3B, Figure S3). Cells transfected with pSARM-GagGFP-M100A alone showed a large delay in release kinetics, and even after a 4 hour chase, only 45% had been released. For cells cotransfected at a ratio of 4:1 D26N to pSARM-GagGFP-M100A, which mimics WT protein expression, in which Pr95 and Pr180 are translated 15% and 5% of the time respectively through ribosomal slippage [25,26], there was an increase kinetics of Gag-GFP release, with 50% of Gag released approximately 3 hours after the pulse (Figure 3A, lane 3; Figure 3B). TEM microscopy of these co-transfected cells revealed capsids with WT morphology (Figure S2C)

In order to confirm that co-assembly of Gag-GFP and untagged Gag was occurring in co-transfected cells, two different experiments were performed. In the first cells were either singly transfected with pSARM-GagGFP-M100A, or co-transfected with this vector and WT pSARM-X , which expresses a functional viral protease. A Western blot analysis, using a mouse anti-GFP antibody, of virions pelleted through a 25% sucrose cushion showed that, in contrast to the 110 kD Gag-GFP released from singly transfected cells, the Gag-GFP in virions from co-transfected cells was efficiently cleaved to the expected p4-GFP (Figure S4, pSARM-GagGFP + WT). 

In the second approach, supernatants from cells transfected with pSARM-GagGFP-M100A or co-transfected with pSARM-D26N, and radiolabeled with ^35^S methionine, were analyzed on discontinuous sucrose gradients. As can be observed in Figure S5, Pr78 and Gag-GFP from co-transfected cells were observed primarily in fractions 4 and 5 (density 1.12 g/mL and 1.17 g/mL). Similar results were obtained for virus released from cells singly transfected by pSARM-D26N (Pr78) and pSARM-X (p27 and gp70). In contrast, virions released from pSARM-GagGFP-M100A transfected cells exhibited a more heterogeneous density and were observed in fractions 3-6 (density 1.08 g/mL - 1.20 g/mL).

Because of these results, further experiments with the pSARM-Gag-GFP-M100A vector included co-transfection with WT pSARM-X at a ratio of 4:1 (WT: GagGFP), or transfection into CMMT cells, a rhesus macaque mammary tumor fibroblast cell line that constitutively expresses WT M-PMV. 

### M-PMV GagGFP subcellular localization is similar to untagged M-PMV provirus

To verify that the GFP fusion was not affecting cellular localization of Gag, COS-1 cells were transfected with untagged forms of wild type provirus or previously described M-PMV Gag mutants (R55W, R22A, K16A/K20A) and following fixation were stained with a rabbit anti-Gag antibody. The staining pattern was compared to COS-1 cells co-transfected with pSARM-GagGFP-M100A or the same vector into which the R55W, R22A, or K16A/K20A mutations were introduced, along with the respective pSARM provirus at a 1:4 ratio. 

The COS-1 cells producing untagged forms of Gag were fixed 24 h after transfection and immunostained; the cells producing GFP fused forms of Gag were fixed 24h after transfection.

**Figure 4:**
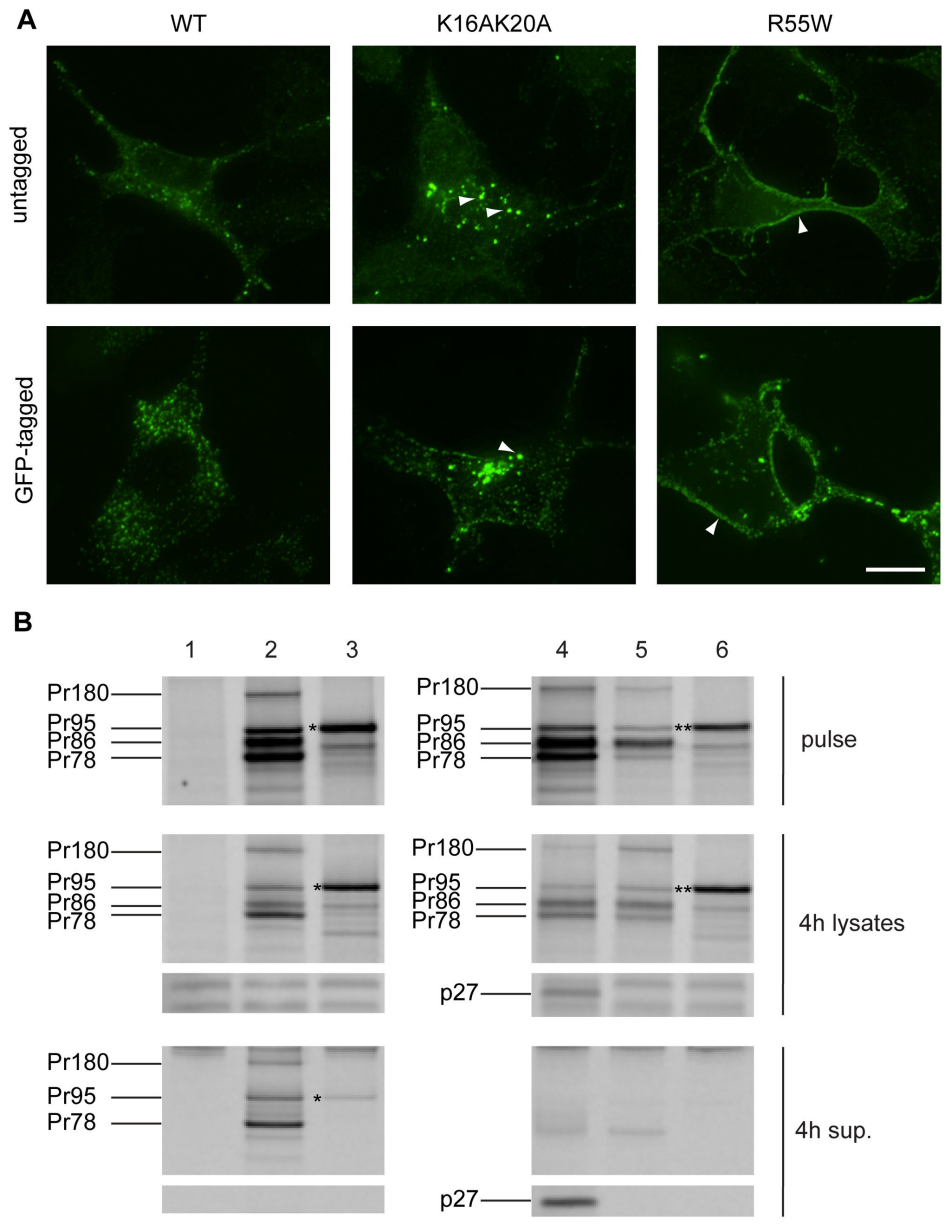
Comparison of untagged and GFP-tagged M-PMV Gag wild-type and mutants in COS-1 cells. (A) COS-1 cells were cotransfected at the ratio of 4:1 with pSARM-WT and pSARM-GagGFP-M100A, pSARM-R55W and pSARM-GagGFP-M100A-R55W, or pSARM-K16A/K20A and pSARM-GagGFP-M100A-K16A/K20A (tagged) or transfected with the untagged pSARM-WT, pSARM-R55W or pSARM-K16A/K20A mutants (untagged). Cells were fixed 24 h post-transfection with cold methanol:acetone (1:1). Cells transfected with untagged provirus were stained by primary rabbit antibody against M-PMV p27 and secondary FITC-conjugated anti-rabbit antibody. Localization of K16A/K20A mutant into intracellular vesicles and localization of R55W on plasma membrane is shown. Scale bar, 20 µm. (B) COS-1 cells (untransfected, Lane 1) were transfected with pSARM-D26N (Lane 2), pSARM-GagGFP-M100A (Lane 3), pSARM-X (Lane 4), pSARM-R22A (Lane 5) and pSARM-GagGFP-M100A-R22A (Lane 6). Viral proteins were metabolically labeled with [^35^S] and then immunoprecipitated with goat anti-MPMV antibody from lysates of pulse-labeled cells (pulse), pulse-labeled and 4h chased cells (4h lysates) and from the culture medium collected after the 4h chase (4h sup.). Positions of the viral precursor proteins Pr180 (Gag-Pro-Pol), Pr95 (Gag-Pro), Pr86 (Envelope) and Pr78 (Gag) are shown. Products of pSARM-GagGFP-M100A (*) and pSARM-GagGFP-M100A-R22A (**) are shown.

Previous work with immunostained wild-type Gag reported a dispersed distribution of Gag in the cytoplasm, with a higher concentration of signal in pericentriolar region, which is the site of particle assembly and Env recruitment [2,10,19]. Similar subcellular localization was observed here in COS-1 cells expressing both untagged (Figure 4A, WT untagged) and WT GagGFP (Figure 4A, WT GFP-tagged). However, the WT GagGFP displayed a somewhat denser staining pattern, likely owing to enhanced protein production resulting from the codon optimization of gag. 

Stansell et al. showed that a double mutation in the matrix domain, K16A/K20A results accumulation of capsids on and efficient budding into intracellular vesicles, with a concomitant 50 to 70% reduction in the efficiency of viral particle release [5]. In both the native and GFP-tagged systems, this mutation resulted in fewer discrete fluorescent capsids in the cytoplasm and the presence of large brightly fluorescent bodies, consistent with the previously described phenotype of budding into intracellular vesicles (Figure 4A, K16A/K20A Untagged and GFP-tagged). Co-staining with the red membrane dye, DiI, supports the conclusion of budding into vesicles (data not shown). 

The R55W mutation within the matrix domain of M-PMV Gag, is known to significantly reduce intracellular assembly and redirect it to the plasma membrane of the cell [3]. In the immunostained cells, the dispersed intracytoplasmic staining of capsids was drastically reduced and was replaced by an intense fluorescent signal along the plasma membrane (Figure 4A, R55W Untagged). A similar, but more intense staining pattern was observed in the Gag-GFP expressing cells (Figure 4A, R55W, GFP-tagged). 

Stansell et al also showed that an R22A mutation in the matrix protein of Gag results in a complete block to virus particle release from infected cells [5]. The particle release defect of the GFP-tagged and untagged form of R22A Gag was verified in a pulse-chase experiment. The cells were pulsed for 30min with ^35^S- methionine and then chased for 4h. M-PMV proteins were immunoprecipitated, using a goat anti-MPMV antibody, then analyzed on an SDS-PAGE gel, from both the pulse (Figure 4B, Pulse gels) and pulse-chase cell lysates (Figure 4B, 4h lysates), as well as from the cell culture medium of the 4h chased cells (Figure 4B, 4h sup). Control wells were transfected with WT-D26N (Figure 4B, lane 2), pSARM-GagGFP-M100A (Figure 4B, lane 3), and pSARM-X (Figure 4B, lane 4), and detectable amounts of Pr78, Gag-GFP, and p27 respectively were observed in the 4h supernatants. In contrast, while pSARM-R22A (Figure 4B, lane 5) and pSARM-GagGFP-M100A-R22A (Figure 4B, lane 6) transfected cells exhibited similar precursor protein patterns in the pulse and pulse-chase cell lysates to their WT counterparts, no released proteins (p27 or Gag-GFP) were detectable in the 4h-supernantants. 

### Real-time imaging of the Gag-GFP fusion construct shows lateral movement throughout the cell

**Figure 5:**
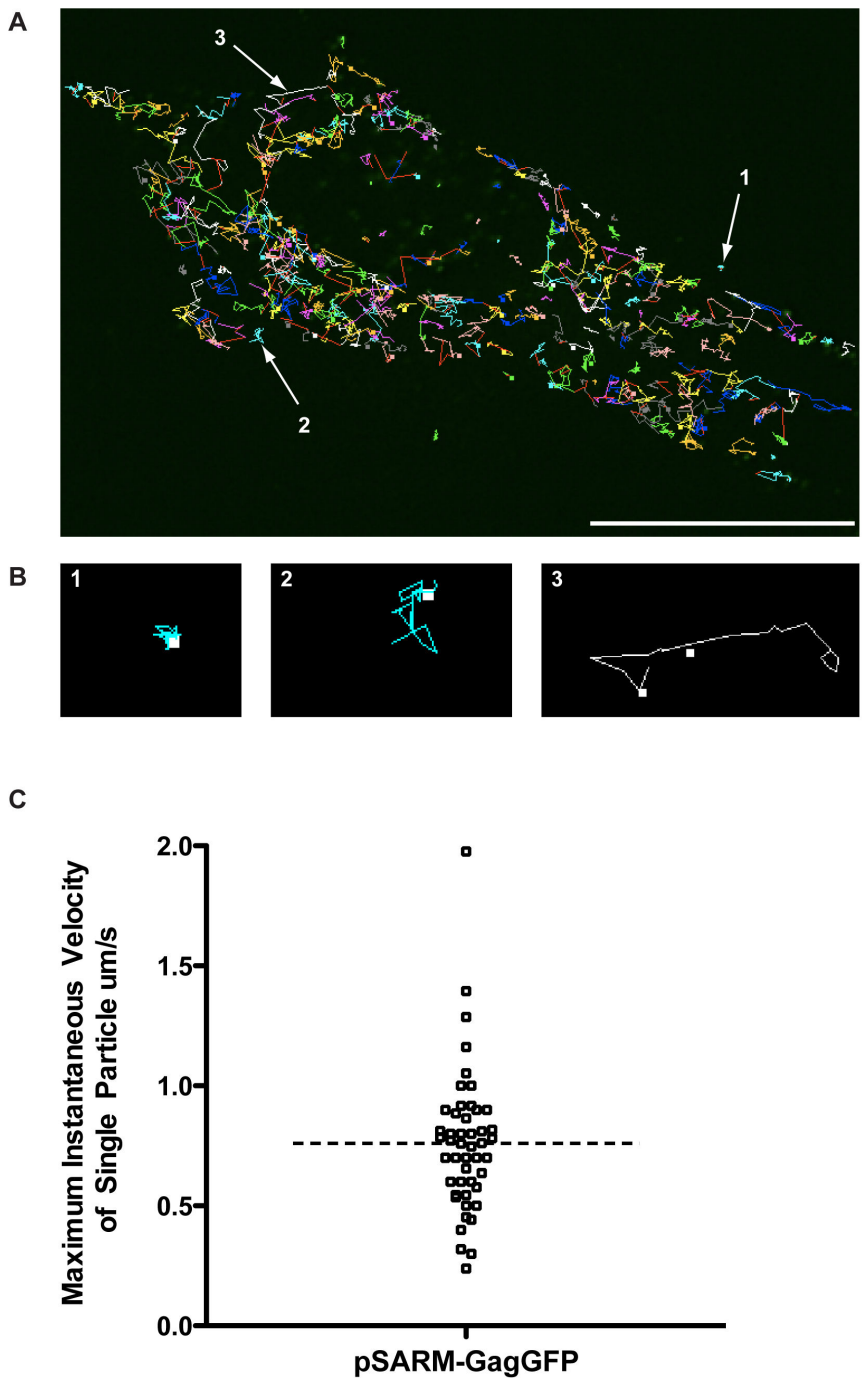
Real-time imaging of GagGFP fusion protein in live cells. (A) M-PMV expressing CMMT cells were transfected with pSARM-GagGFP-M100A and visualized 24 hours later in real-time every 5 seconds for 2 minutes. Using the Particle Tracker plug-in on FIJI, particles were detected and then tracked. Scale bar = 20 µm. Real time imaging of these frames is shown in movie S1. (B) Trajectory data showed trajectories with restricted movement and little to no displacement from the beginning to the end of the trajectory path (1), trajectories where the particle moved in an erratic, Brownian manner, and displayed very little displacement (2) and long tracks with significant displacement from the beginning of the track to the end (3). (C) To calculate the instantaneous velocity for a trajectory in population 3, the distance traveled between each time point was calculated and divided by the time interval (5s). Maximum instantaneous velocity refers to the maximum instantaneous velocity for an individual trajectory.

M-PMV-expressing CMMT cells were transfected with pSARM-GagGFP-M100A and visualized in real-time with exposures every 5 seconds for 2 minutes. Using the Particle Tracker plugin on the FIJI software package, particles were detected and then automatically tracked [24].Trajectory data showed three different populations of fluorescent particles (Figure 5A, Movie S1, Figure S6). Population 1 showed trajectories with restricted movement and little to no displacement from the beginning to the end of the trajectory path (Figure 5B.1). The median displacement for trajectories that are seen in this population is 1.6 pixels, and occurred approximately 15% of the time for trajectories containing at least 10 time points (Figure S6A and B). Population 2 showed particles moving in an erratic, Brownian manner, with very little displacement (Figure 5B.2). The trajectories in this population appear larger than those of the first population, although there is very little long-range movement within the trajectory. The median total displacement of particles in this population is significantly higher (6.9 pixels) than in Population 1. Tracks in this population made up approximately 41% of the trajectories that had at least 10 times points (Figure S6A and B). Population 3, occurring 44% of the time, displayed long, linear tracks with, in most cases, significant displacement from the beginning of the track to the end (Figure 5B.3). The median displacement in this population was 15.5 pixels (Figure S6B). This lateral movement across the cell was bidirectional in nature at times, and the instantaneous velocities oscillated between periods of complete cessation of movement followed by periods of long-range movement. The median, maximum instantaneous velocity of tracks in Population 3 was approximately 700nm/s (Figure 5C). 

### Fluorescently-labeled capsids associate and transport along microtubules

**Figure 6:**
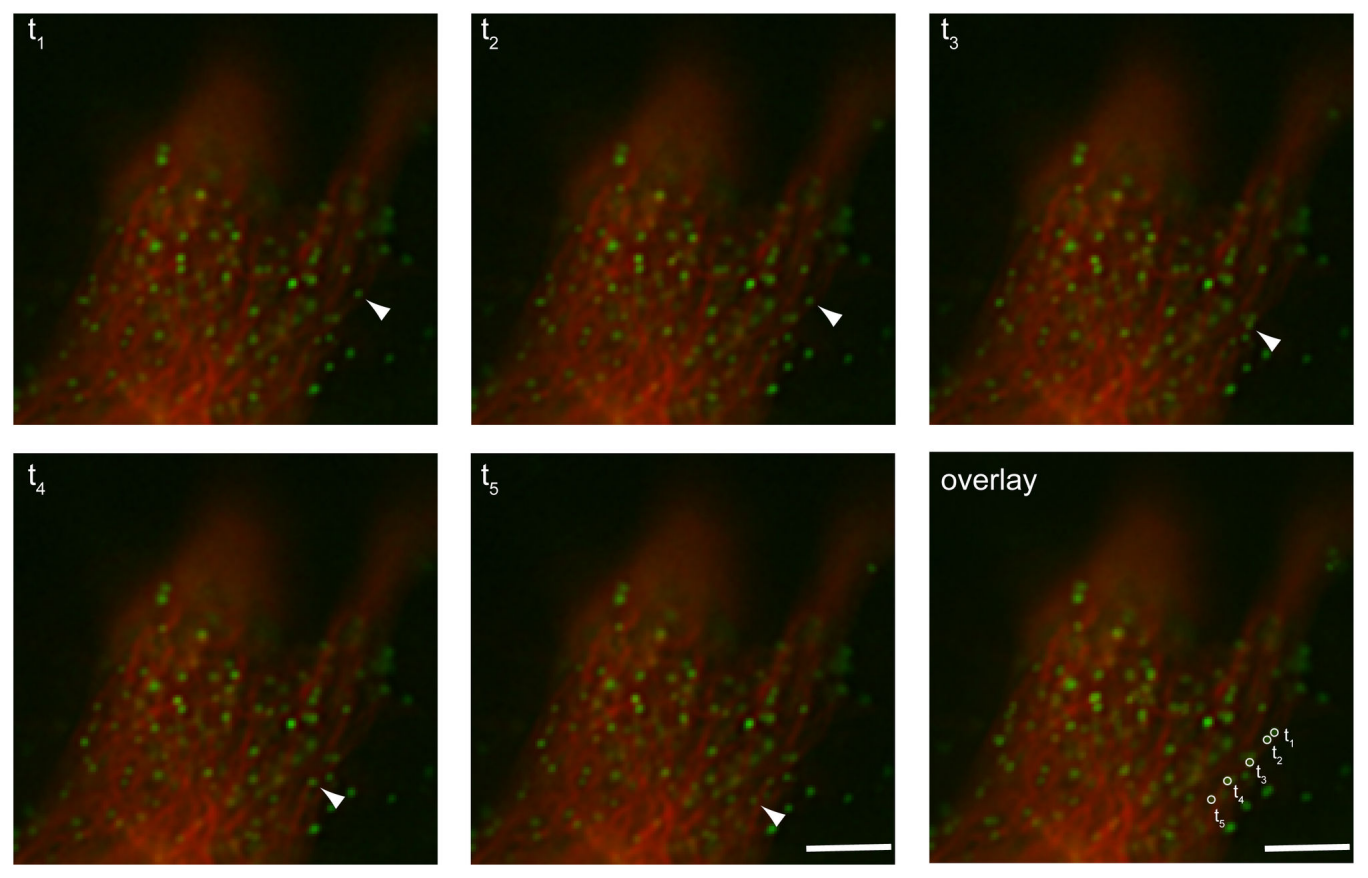
Real-time imaging of GFP labeled virus-like particles along the microtubule. M-PMV expressing CMMT cells were cotransfected with pSARM-GagGFP-M100A and mCherry-tubulin and imaged 24 hours later in real-time. Images were captured at consecutive intervals of 1 second for one minute in one focal plane. Scale bar= 1 µm. Real time imaging of these frames is shown in movie S2.

The results from the live cell imaging of capsid transport suggested a possible role for microtubules in their intracellular transport. Specifically, the bidirectional movement and the oscillating velocities of capsid movement have been previously described for endosomal movement as well as viral movement along microtubules [27-30]. To test whether the capsids were associated with and were transported along microtubules, CMMT cells were co-transfected with pSARM-GagGFP-M100A and mCherry-labeled tubulin and then imaged 24 hours post-transfection. Images were captured every second for one minute in one z-stack. Time-lapse data of these cells shows a majority of the capsids are not moving, but nevertheless colocalize with a tubule (Movie S2). When a smaller section of the cell is examined at higher effective magnification, capsids that display rapid, lateral movement with significant displacement appear to traffic along the path of the microtubules (Figure 6). Examples of retrograde and anterograde movement along tubules can be seen, as well as examples of capsid that appear to switch from one tubule to another (data not shown). 

## Discussion

To date, the host cell factors involved in the assembly and intracellular trafficking of retroviral proteins remain poorly understood. For M-PMV, much work has been done to elucidate the cellular factors involved in assembly of premature capsids in the pericentriolar region of the cell. Factors such as the microtubule organizing center and dynein motors have previously been described to interact with the Gag assembling on polysomes through an interaction with the cytoplasmic targeting and retention signal in the matrix domain of Gag. It has also been shown that the cellular vesicular trafficking system has a role in intracellular capsid transport, since a temperature block of 20°C, which reversibly inhibits vesicle trafficking, causes an accumulation of capsid particles at the pericentriolar site of assembly [2,7,10,12]. In addition, several mutations in the cytoplasmic domain of the Env glycoproteins [2,7,10,12], and others in the Gag polyprotein has suggested that endocytic recycling pathways are critical for Env’s involvement in efficient capsid intracellular transport [7,10]. However, most of these studies utilized population-based pulse-chase experiments and fixed cell immunofluorescence. The availability of fluorescently labeled M-PMV proteins that can be visualized in a living cellular system clearly has the potential to provide more insight into the roles of cellular factors in the intracellular transport of M-PMV capsids from their site of assembly to the plasma membrane site of egress. 

### Constructing and optimizing an M-PMV Gag-GFP fusion

The goal of this study was to construct an M-PMV Gag-GFP fusion protein that could be visualized in live cells and to use this construct to investigate intracellular trafficking of M-PMV capsids. First, the *gag*-gene of M-PMV was codon optimized to prevent aberrant splicing observed with the native coding region. Despite this, fusion of *gfp* to the 3’ end of the codon-optimized *gag* resulted in the production of a truncated GFP-tagged protein, in addition to the full-length product. This shorter product was shown to result from ribosomal initiation from an internal, in-frame methionine 100 amino acids from the primary initiation codon for *gag*. Although this methionine is present in the WT M-PMV *gag*, only background levels of protein are initiated from this methionine. However, previous work had shown that even single nucleotide changes to the second codon of Gag could result in ribosomes skipping the initiating methionine (M1) and initiating translation of a truncated Gag more than 50% of the time [31]. It is therefore probable that initiation from this second methionine in the GFP fusion construct was due to codon optimization, which allowed the ribosome to recognize this second methionine at a higher rate than it would in the non-codon optimized *gag*. Introduction of the M100A mutation, previously shown to have no effect on virus infectivity, and optimization of the Kozak sequence before M1, resulted primarily in full-length translation products. 

Another caveat to constructing a Gag-GFP fusion protein that is biologically relevant is the fact that fusion of a 27-kD GFP protein to the carboxyl-terminus of Gag could interfere with capsid morphogenesis. Indeed in a previous study, only incomplete and aberrant particles were assembled when the frame-shifting signal at the end of *gag* was mutated to generate only Gag-Pro and Gag-Pro-Pol precursors [32]. When 293T cells were transfected with the pSARM-Gag-GFP-M100A construct alone, transmission electron microscopy showed the assembly of larger, non-spherical capsids that had an unusual “beads-on-a-string” external surface. Additionally, when virus was subjected to density centrifiguation through a 20%-50% sucrose gradient, GagGFP-containing virions appear in fractions 3-6, while WT virions and virions from co-transfected cells were primarily present in the 4^th^ and 5^th^ fractions, confirming that virions from cells transfected with pSARM-GagGFP-M100A alone have a more diverse and in some cases lower density than WT virions. Moreover, pulse-chase analyses showed that the kinetics of release for these aberrant capsids was also greatly reduced. In order to assemble a GFP-tagged capsid that is transported with kinetics similar to a WT capsid, co-assembly of Gag and Gag-GFP proteins in ratios resembling Gag and Gag-Pro must occur. Because of this, all experiments involving the Gag-GFP fusion protein were performed in the context of WT virus, either by cotransfection or by transfection of Gag-GFP into the CMMT cell line that constitutively expresses WT M-PMV. 

To confirm further that that the Gag-GFP fusion protein behaved similarly to untagged, WT Gag, a comparison was made of the subcellular localization of both WT and several well-characterized Gag mutants. Immunofluorescent staining showed that GFP-tagged Gag localized similarly to the untagged Gag. WT forms of both exhibited a dispersed distribution of fluorescent puncta, with a concentration in the pericentriolar region. This pattern was dramatically altered in pSARM-GFP-M100 vectors encoding the R55W and K16A/K20A mutants. In the former, Gag staining outlined the periphery of the cells with predominantly diffuse staining in the cytoplasm, consistent with capsid assembly now occurring on the plasma-membrane [3]. And for the latter, brightly staining vesicular structures were observed, consistent with budding into a variety of intracellular vesicles [5]. Along with this visual evidence that the Gag-GFP fusion protein behaves similarly to untagged Gag, a pulse-chase analysis of the tagged and untagged R22A mutant showed that, like the untagged mutant, pSARM-GagGFP-M100A-R22A also failed to be released from cells. This provides compelling evidence that the Gag-GFP fusion protein utilizes virus-cell interactions comparable to the untagged Gag, and can be used for characterizing the nature of M-PMV Gag intracellular transport in dynamic, real time applications. 

### Evidence for M-PMV capsid use of the cellular cytoskeleton for transport

Particle tracking of GFP-tagged capsids in transfected CMMT cells and COS-1 cells provided evidence for three types of capsid movement. In the first, the particles exhibited very little lateral movement, consistent with them being anchored to a subcellular structure. In the second, capsid movement was consistent with Brownian movement – presumably representing capsids free in the cytoplasm. Finally, a subset of capsids exhibited long-range oscillatory movement, with kinetics that are consistent with movement on microtubules. Characterization of endosomal intracellular transport, as well as the interaction of several different viruses with the microtubular network of the cell, shows that microtubular cargo have instantaneous velocities that are oscillatory in nature and movement is bidirectional [27-30,33]. The microtubule motors involved in anterograde transport toward the plasma membrane, kinesins, have been shown to propel cargo as fast as 4 μm/s, as well as to stall cargo during “pauses” [27-29,33,34]. Microtubule-dependent transport of viruses has been extensively studied in Vaccinia virus [30,35]. In these studies, intracellular enveloped viruses (IEV) have been shown to require the use of microtubule motor, kinesin-1, for saltatory transport from their intracellular site of replication and assembly to the cortical actin layer. These studies have shown a wide range of instantaneous velocities, with an average of approximately 8 µm/s. Live cell imaging analysis from these studies also describe bidirectional movement of IEVs and movement consistent with switching of IEVs from one microtubule to another.

In this current study, a subset of capsids was observed to be associated with microtubules, but not moving. Since these kinetic studies capture only a very small fraction of time (1-2 minutes), it is possible that the microtubule-associated capsids are stalled along the microtubule at this time. The capsids that appear to be transporting along the microtubules have instantaneous maximum velocities up to 2 µm/s with a median of 700 nm/s, when images were captured every 5 seconds. The capsid movement observed in our system also displays bidirectional transport, and evidence of jumping from one microtubule to another microtubule can also be seen. These observations provide strong support for the hypothesis that the capsids are utilizing microtubules for intracellular transport. 

A major caveat to this study is that the real-time imaging only captures approximately 4% of the time necessary for 50% of capsids to be released from the cell. This means the imaging is limited to characterizing localized movement of individual capsids in a small window of time. Further experimentation must be performed to characterize the collective nature of capsid movement over longer periods of time. 

## Conclusion

The current study shows that employing the use a fluorescently-tagged M-PMV Gag protein can be useful in characterizing the nature of M-PMV Gag assembly and transport in a real-time imaging system. In order to utilize this system, however, it is necessary to use a codon-optimized Gag with an M100A mutation in the MA protein to prevent internal initiation. Codon-optimization results in a dysfunction in splicing necessary for Env expression, and Env expression has been shown to be critical in efficient capsid release kinetics. TEM results also show that transfection of 293T cells with the Gag-GFP fusion protein results in aberrantly shaped capsids. Both of these phenotypes amplify the importance of co-expression of the Gag-GFP fusion protein with the WT or D26N Gag from a proviral vector, which both express Env at WT levels and allows for co-assembly for tag and untagged Gag proteins. Following optimization of these conditions, Gag-GFP expressing cells display intracellular movement of assembled capsids that is consistent with transport along microtubules. 

## Supporting Information

Figure S1 **Annotated nucleotide and amino acid sequence of pSARM-GagGFP-M100A.**
The nucleotide sequence for pSARM-GagGFP-M100A was imported into the bioinformatics software Geneious^TM^ version 5.5.5 (Biomatters, www.geneious.com). With this software, the nucleotide sequence was translated in the correct frame. The position of the optimized Kozak consensus sequence, the initiating methionine (M1), the methionine to alanine substitution (M100A), GA-linker used to separate Gag and eGFP, and two putative splice acceptor sites for Env were annotated.
(PDF)

Figure S2 **Transmission electron microscopic imaging of the intracellular localization of immature** **capsids**.Transmission electron microscopy (TEM) images of COS cells transfected with pSARM-X (A), pSARM-GagGFP-M100A (B), and cotransfected with pSARM-X and pSARM-GagGFP (C). Arrowheads point to representative immature M-PMV capsids.
(TIFF)

Figure S3 **Percent GagGFP released from cells.** Quantitation of the % GagGFP released from the cell at each time point from pulse-chase analysis of 293T cells transfected with pSARM-GagGFP-M100A only (red), a 4:1 ratio of pSARM-D26N and pSARM-GagGFP-M100A as compared to the total amount of Gag released from cells transfected with pSARM-D26N (blue).
(TIFF)

Figure S4 **Western blot analysis of virus released from cotransfected cells.** (A) Western blot of supernatants from 293T cells, untransfected (lane 1), or transfected with pSARM-GagGFP-M100A only (lane 2) or a 4:1 ratio of pSARM-X and pSARM-GagGFP-M100A (lane 3). Supernatants were resolved on 12% SDS-PAGE, and blotted with antibody against GFP. (B) Quantitation of band intensity of western blot. Black bars represent the band corresponding to the uncleaved Gag-GFP fusion and white bars represent the cleaved p4-GFP found in 293T cells transfected with pSARM-GagGFP-M100A, or cotransfected with pSARM-GagGFP-M100A and pSARM-X.
(TIFF)

Figure S5 **Density fractionation of virions released into the cell supernatant.** (A) Sucrose gradient density fractionation of supernatants from 293T cells cotransfected with pSARM-D26N and pSARM-GagGFP, pSARM-GagGFP alone, pSARM-X, pSARM-D26N, or untransfected (mock). Culture supernatants were collected 48 hours after transfection and overlayed on a 20%-50% (w/w) sucrose gradient, followed by ultra-centrifugation in at 35,000 rpm for 3 hours in a SWTi-41 rotor. Fractions were collected by upward displacement and immunoprecipitated with an antibody against whole M-PMV. Samples were resolved on 12% SDS-PAGE gel. (*) represents the band corresponding to Gag-GFP fusion protein. (B) The refractive index of each fraction was measured using a refractometer. The density was determined by comparing the refractive indices to a standard conversion table for the density and refractive index in sucrose.
(TIF)

Figure S6 **(**A**) Percentage of each type of trajectory found in Figure 5A.** Analysis is based on categorizing 20 different trajectories from 4 subsections of the cell (left of nucleus, above nucleus, below nucleus, and right of nucleus). (B) The total displacement of 10 separate trajectories from each population type was calculated. The line represents the median for each population. Statistical analysis was based on the non-parametric Mann-Whitney t-test.
(TIFF)

Movie S1 **Real-time imaging of GagGFP fusion in CMMT cells.** M-PMV expressing CMMT cells were transfected with pSARM-GagGFP-M100A and visualized on the Deltavision Core Imaging System 24 hours later. 3D images (with 10 z-sections spaced 200 nm apart) were in captured every 5 seconds for a total of 2 minutes. The video is a projection of all z-sections in one plane.
(AVI)

Movie S2 **Real-time imaging of GFP labeled virus-like particles along the microtubule.** M-PMV expressing CMMT cells were cotransfected with pSARM-GagGFP-M100A and mCherry-tubulin and imaged 24 hours later in real-time. Images were captured at consecutive intervals of 1s for one minute in one focal plane.
(AVI)
