# Supplementary figures and images for: A Mason-Pfizer Monkey Virus Gag-GFP Fusion Vector Allows Visualization of Capsid Transport in Live Cells and Demonstrates a Role for Microtubules
Source: PLoS One. 2013 Dec 26;8(12):e83863. doi: 10.1371/journal.pone.0083863 (PMC3873405; doi:10.1371/journal.pone.0083863)

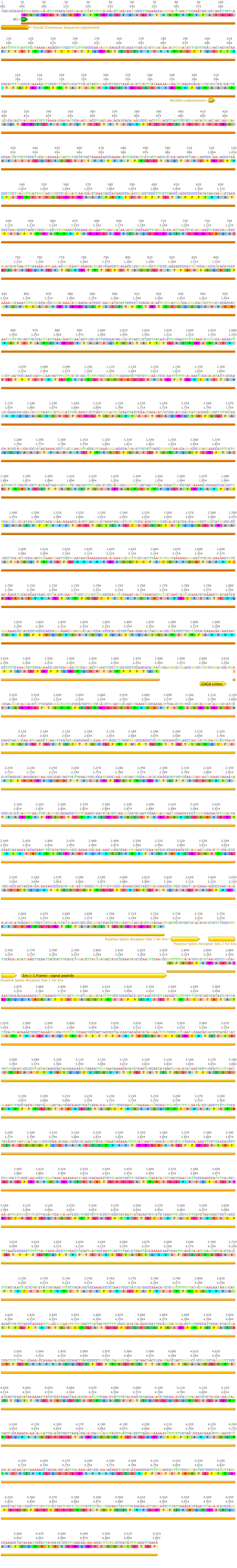

Supplement: Figure S1 — Annotated nucleotide and amino acid sequence of pSARM-GagGFP-M100A. The nucleotide sequence for pSARM-GagGFP-M100A was imported into the bioinformatics software GeneiousTM version 5.5.5 (Biomatters, www.geneious.com). With this software, the nucleotide sequence was translated in the correct frame. The position of the optimized Kozak consensus sequence, the initiating methionine (M1), the methionine to alanine substitution (M100A), GA-linker used to separate Gag and eGFP, and two putative splice acceptor sites for Env were annotated. (PDF) [file pone.0083863.s001.pdf]

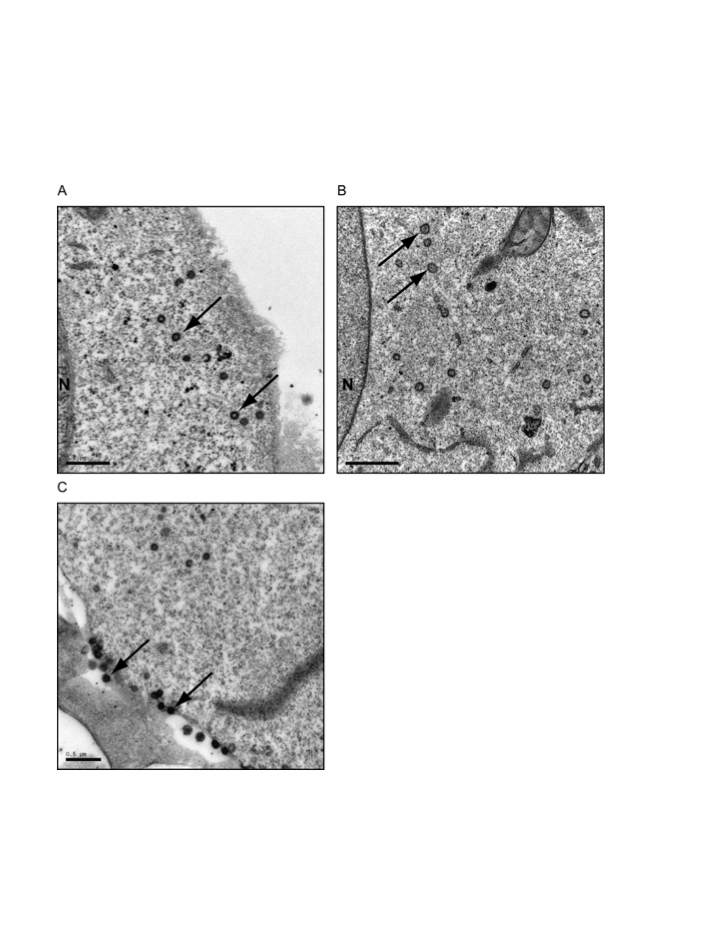

Supplement: Figure S2 — Transmission electron microscopic imaging of the intracellular localization of immature capsids.Transmission electron microscopy (TEM) images of COS cells transfected with pSARM-X (A), pSARM-GagGFP-M100A (B), and cotransfected with pSARM-X and pSARM-GagGFP (C). Arrowheads point to representative immature M-PMV capsids. (TIFF) [file pone.0083863.s002.tiff]

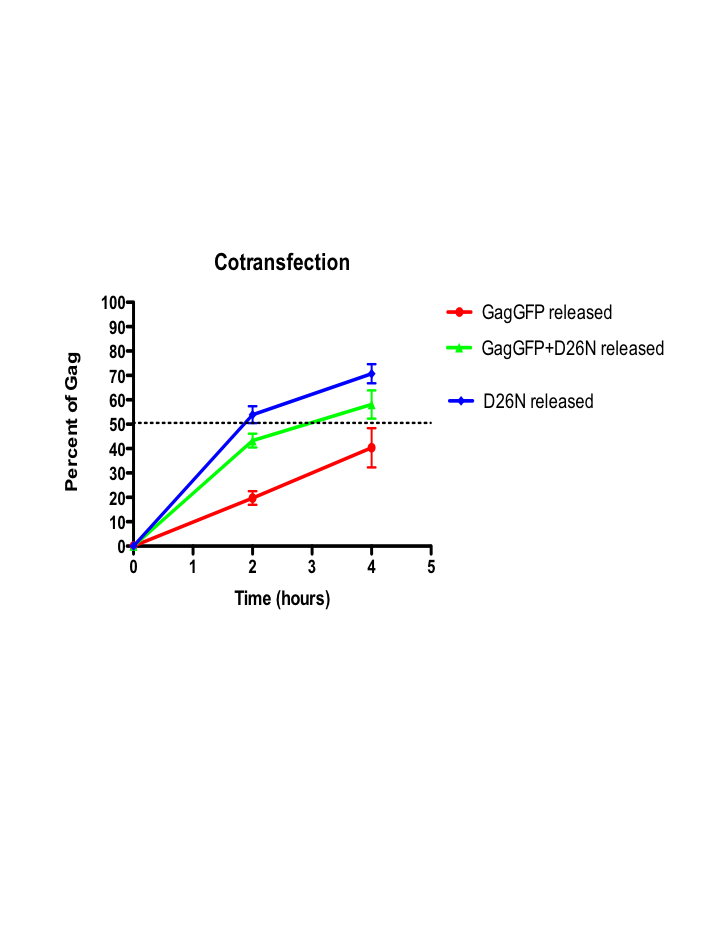

Supplement: Figure S3 — Percent GagGFP released from cells. Quantitation of the % GagGFP released from the cell at each time point from pulse-chase analysis of 293T cells transfected with pSARM-GagGFP-M100A only (red), a 4:1 ratio of pSARM-D26N and pSARM-GagGFP-M100A as compared to the total amount of Gag released from cells transfected with pSARM-D26N (blue). (TIFF) [file pone.0083863.s003.tiff]

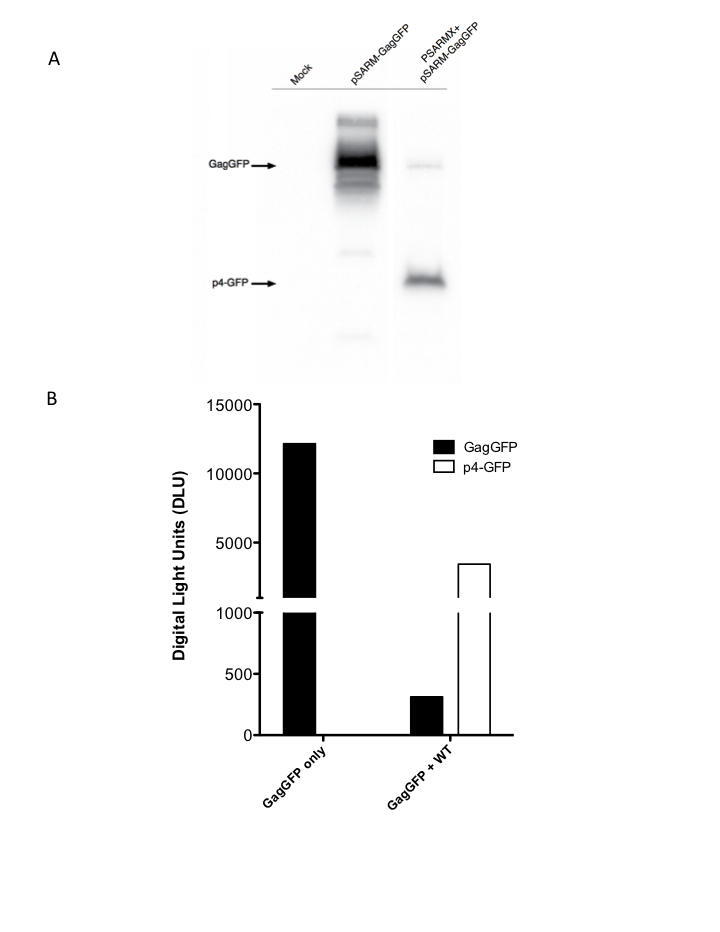

Supplement: Figure S4 — Western blot analysis of virus released from cotransfected cells. (A) Western blot of supernatants from 293T cells, untransfected (lane 1), or transfected with pSARM-GagGFP-M100A only (lane 2) or a 4:1 ratio of pSARM-X and pSARM-GagGFP-M100A (lane 3). Supernatants were resolved on 12% SDS-PAGE, and blotted with antibody against GFP. (B) Quantitation of band intensity of western blot. Black bars represent the band corresponding to the uncleaved Gag-GFP fusion and white bars represent the cleaved p4-GFP found in 293T cells transfected with pSARM-GagGFP-M100A, or cotransfected with pSARM-GagGFP-M100A and pSARM-X. (TIFF) [file pone.0083863.s004.tiff]

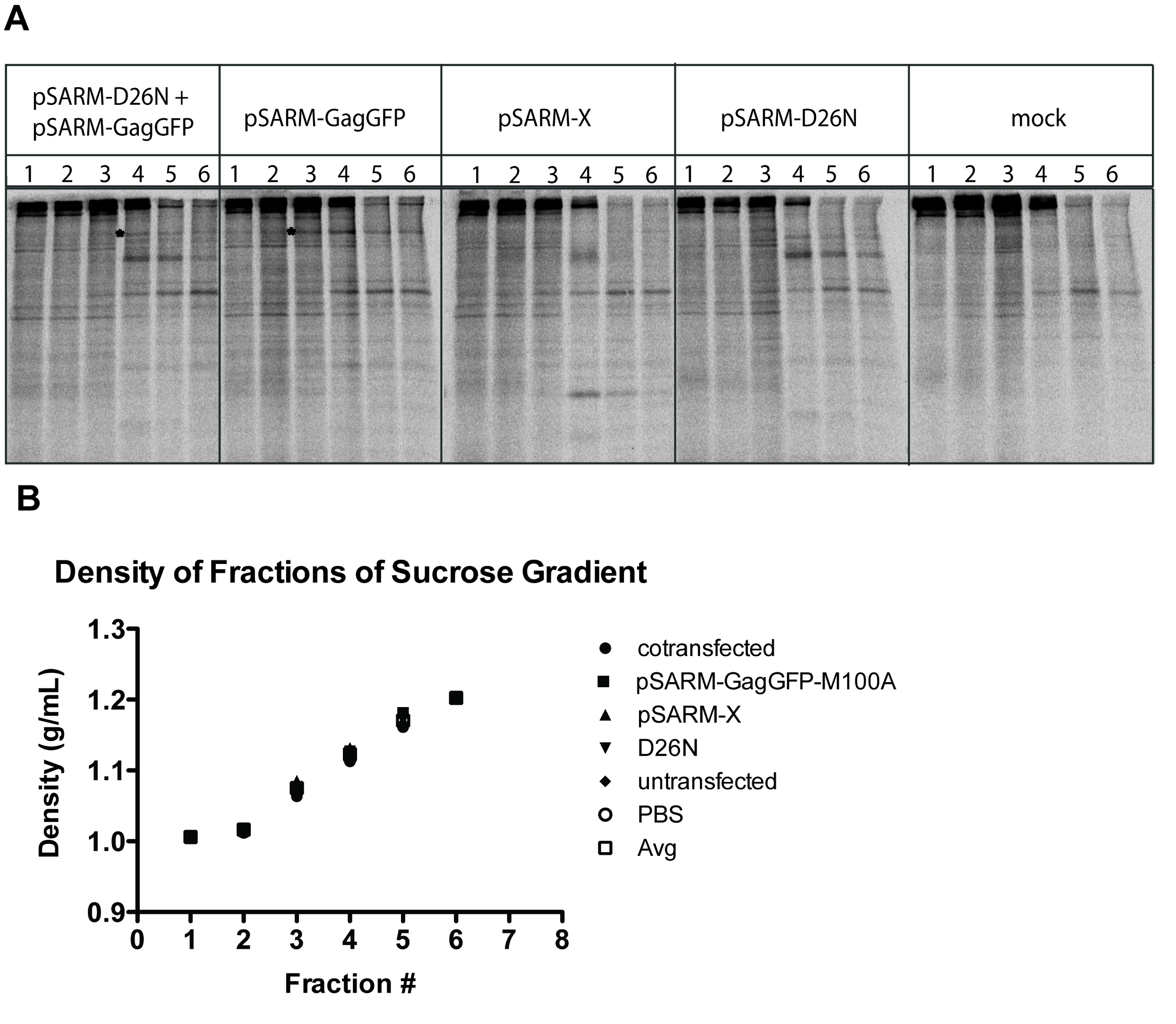

Supplement: Figure S5 — Density fractionation of virions released into the cell supernatant. (A) Sucrose gradient density fractionation of supernatants from 293T cells cotransfected with pSARM-D26N and pSARM-GagGFP, pSARM-GagGFP alone, pSARM-X, pSARM-D26N, or untransfected (mock). Culture supernatants were collected 48 hours after transfection and overlayed on a 20%-50% (w/w) sucrose gradient, followed by ultra-centrifugation in at 35,000 rpm for 3 hours in a SWTi-41 rotor. Fractions were collected by upward displacement and immunoprecipitated with an antibody against whole M-PMV. Samples were resolved on 12% SDS-PAGE gel. (*) represents the band corresponding to Gag-GFP fusion protein. (B) The refractive index of each fraction was measured using a refractometer. The density was determined by comparing the refractive indices to a standard conversion table for the density and refractive index in sucrose. (TIF) [file pone.0083863.s005.tif]
